# Aspirin Promotes Oligodendrocyte Precursor Cell Proliferation and Differentiation after White Matter Lesion

**DOI:** 10.3389/fnagi.2014.00007

**Published:** 2014-01-27

**Authors:** Jing Chen, Shilun Zuo, Jing Wang, Jian Huang, Xiao Zhang, Yang Liu, Yunxia Zhang, Jun Zhao, Junliang Han, Lize Xiong, Ming Shi, Zhirong Liu

**Affiliations:** ^1^Department of Neurology, Xijing Hospital, Fourth Military Medical University, Xi’an, China; ^2^Department of Neurosurgery, Southwest Hospital, Third Military Medical University, Chongqing, China; ^3^Department of Anesthesiology, Xijing Hospital, Fourth Military Medical University, Xi’an, China

**Keywords:** aspirin, oligodendrocytes, oligodendrocyte precursor cells, white matter lesion, extracellular signal-related kinase, RhoA

## Abstract

Cerebral white matter lesion (WML) is one of the main causes for cognitive impairment and is often caused by chronic cerebral hypoperfusion. A line of evidence has shown that aspirin has neuroprotective effects and produces some benefits in long-term outcome and survival for ischemic stroke patients. However, whether aspirin exerts a protective effect against WML is still largely unknown. Here, we showed that aspirin could promote oligodendrocyte precursor cell (OPC) proliferation and differentiation into oligodendrocytes after WML. Male Sprague-Dawley rats were subjected to permanent bilateral common carotid artery occlusion, a well-established model for WML. Four weeks later, Morris water maze test showed an impairment of learning and memory ability of rat while aspirin treatment improved behavioral performance. Low dose of aspirin (25 mg/kg) was found to elevate the number of OPCs while relatively high doses (100–200 mg/kg) increased that of oligodendrocytes, and ameliorated WML-induced the thinning of myelin, as revealed by the electron microscope. Similarly, our *in vitro* study also showed that relatively low and high doses of aspirin enhanced OPC proliferation and differentiation into oligodendrocytes, respectively. Furthermore, we revealed that aspirin enhanced extracellular signal-related kinase (ERK) but inhibited RhoA activities. In summary, we provided the first evidence that aspirin can promote oligodendrogenesis and oligodendrocyte myelination after WML, which may involve ERK and RhoA pathways.

## Introduction

Cerebral white matter lesions (WML) are observed in aging and stroke and constitute the core pathology of Binswanger disease, a form of subcortical vascular dementia (Shibata et al., 2004). These WML are believed to be responsible for cognitive impairment and are caused by chronic cerebral hypoperfusion (Pantoni and Garcia, 1997). There is an evidence suggesting that the cerebral white matter is as vulnerable to ischemia as cerebral gray matter (Pantoni et al., 1996). Dewar et al. (1999) emphasized that total brain protection, in which not only gray matter but also white matter is protected, is important and necessary.

However, damage to white matter, which is composed of myelinated axons and oligodendrocytes, has been largely neglected. Multiple mechanisms were involved in WML. For example, maturation-dependent vulnerability in the oligodendrocyte lineage was found in a hypoxic–ischemic injury model (Back et al., 2002). Oligodendrocytes are best known as the myelin-forming cells in the central nervous system (CNS). Oligodendrocyte precursor cells (OPCs) are immature oligodendrocytes and can differentiate into myelin-forming cells under certain conditions (Fu et al., 2005). In the adult brain, mature myelinating oligodendrocytes are continuously produced from local OPCs residing in the brain parenchyma (Gensert and Goldman, 1997; Fancy et al., 2004) and from precursor cells located in the subventricular zone (Picard-Riera et al., 2002; Fancy et al., 2004; Menn et al., 2006). Given their high migratory potential and their ability to differentiate into myelin-forming cells, subventricular neural stem cells (NSCs) represent an important endogenous source of OPCs for preserving the oligodendrocyte population in the white matter and for the repair of demyelinating injuries (Gonzalez-Perez and Alvarez-Buylla, 2011). Therefore, it is proposed that any drug, which can increase the number of OPCs and/or oligodendrocytes, may be beneficial for the treatment of WML.

Aspirin has become a standard treatment for acute ischemic stroke since it produces some benefit in long-term outcome and survival if given within 14 days of stroke onset (Hankey et al., 2003). Depending on its dosage, aspirin has a wide spectrum of pharmacological activities and multiple sites of action, which may contribute to the neuroprotection (Berger et al., 2004). So far, numerous studies have shown the neuroprotective effects of aspirin (Lorenzo Fernandez, 2002; Castillo et al., 2003; Vartiainen et al., 2003; Berger et al., 2004; Asanuma et al., 2012). For example, aspirin was found to be protective against ischemia-induced neuronal damage in animal model (Castillo et al., 2003; Berger et al., 2004) and in patients (Castillo et al., 2003), and against dopamine quinone-induced neurotoxicity (Asanuma et al., 2012). Based on the evidence above, we proposed that aspirin might exert a protective action against WML. Thus, in this study, using a well-established WML model induced by chronic cerebral hypoperfusion, we examined the effects of different doses of aspirin on rat learning and memory ability and the changes in the expression of oligodendrocyte lineage markers, and further explored underlying mechanisms of aspirin.

## Materials and Methods

### *In vivo* experiments

#### Animals and WML model

Adult male Sprague-Dawley (SD) rats (from the Fourth Military Medical University Laboratory Animals Center, Xi’an, China), weighing 250–300 g, were kept under standard housing conditions at a temperature between 20 and 23°C, with a 12-h light–dark cycle and a relative humidity of 50%. Rats were divided into three groups randomly, the normal group, the control group, and the aspirin group. Rats in the control and aspirin groups were undertaken with WML model induced by permanent bilateral common carotid artery occlusion, as described previously (Farkas et al., 2007). During the operation, the rat rectal temperature was monitored and maintained at 37 ± 0.5°C. Immediately after cerebral ischemia, the rats in aspirin group were received different doses (25, 50, 100, and 200 mg/kg, dissolved in 10% warm ethanol) of aspirin (Sigma-Aldrich, St. Louis, MO, USA) daily for 4 weeks intraperitoneally, and those in the control group were received the same volume of ethanol. All experimental procedures were reviewed and approved by the Animal Studies Committee of Fourth Military Medical University, Xi’an, China and animal study was carried out with the established institutional guidelines regarding animal use and care.

#### Spatial learning and memory test

Morris water maze behavioral test was used to assess animal spatial learning and memory abilities, as described previously (Morris, 1984). During the training trials, the platform location was fixed and submerged under opaque water. Rats in each group (*n* = 5) received four trials per day for four consecutive days. Each cycle consisted of 90 s trials with 10 min in inter-trial interval. The time to reach the platform was recorded every time. Once rats located the platform, they were allowed to remain on it for 30 s. The rats that failed to find the platform within 90 s were manually placed on the platform for 30 s either. The rats that failed during four consecutive days were excluded from the analysis because of the possibility of a visual disturbance. For the assessment of spatial memory retention, a 60-s probe trial was conducted at the end of training, in which the platform was removed from the pool.

#### Anxiety-related behavior tests

Open field test was performed in a square arena (60 cm × 60 cm), with a white floor divided into 36 squares (10 cm × 10 cm), enclosed by continuous, 25 cm-high walls made of black plexiglass. The arena was lit by two red-light lamps (2 × 60 W) placed over its center. In this test, the 20 squares adjacent to the wall represent a protected field, named “arena periphery,” while the other 16 squares represent an exposed field, or “arena center.” The animals were routinely tested during the first half of the dark phase of their light/dark cycle. The test was initiated by placing a single rat in the middle of the arena and allowing it to move freely for 15 min. Rat behaviors were continuously monitored under a video camera, and the total amount of time each rat spent in the arena center was recorded.

Elevated plus test was performed, as described previously (Carola et al., 2002). The test was initiated by placing a rat on the central platform of the maze, facing one of the open arms, and allowing it to move freely. Each session lasted 5 min and rat behavior was monitored under a video camera placed above the apparatus. Open-arm visits, closed-arm visits, and the total visits of rat were recorded.

#### BrdU pulsing

For differentiation assays, the rats were received BrdU (100 mg/kg, i.p. Sigma) once a day for 14 days after operation and then sacrificed at 4 weeks for the observation of newly differentiated oligodendrocytes. For proliferation assays, the rats were received three pulses of BrdU (100 mg/kg) within 24 h before the animals were sacrificed for the observation of proliferating OPCs.

#### Electron microscope

For electron microscopy, the tissues from epon-embedded corpus callosum were trimmed and reoriented so that ultrathin cross sections could be cut and treated with uranyl acetate and lead citrate. Electron micrographs were analyzed using the analysis DocuSystem (Soft Imaging System). At least 800 fibers from each rat (*n* = 3 per treatment) were analyzed. According to a previous study (Coetzee et al., 1996), axon diameter and fiber diameter were measured (axon diameter was defined as the axon diameter excluding myelin, in contrast to fiber diameter, which was defined as axon diameter plus myelin) and *G*-ratios, which were defined as the diameter of the axon divided by fiber diameter, were calculated.

### *In vitro* experiments

#### Neural stem cell culture

Primary NSCs were isolated from the cerebral cortex of embryonic day (E) 14.5 SD rats, as described previously (Chojnacki and Weiss, 2008). The cells grew as free-floating aggregates and were harvested and mechanically dissociated to produce single cell suspension for replating every 4–6 days. After a minimum of three passages, single cell suspensions were plated on poly-l-lysine-coated (Sigma) coverslips at a density of 5 × 10^4^ cells/coverslip, and maintained in a differentiation medium [DMEM/F12 (Gibco/Invitrogen, Carlsbad, CA, USA), 2% B27 (Gibco) and 1% fetal bovine serum (FBS, HyClone, Logan, UT, USA)] with treatment of aspirin (0.1, 0.5, 1, 5, and 10 μM) or same volume of ethanol for 4 or 7 days. After fixed in 4% paraformaldehyde for 30 min, the cells were processed for immunostaining, as described below.

#### OPC culture

Oligodendrocyte precursor cell culture and purification were performed according to a previous study (Niu et al., 2012). For the observation of OPC proliferation, 5 × 10^4^ cells were seeded on coverslips and grew in modified OPC growth-medium (mOGM) containing 15% of B-104CM, 1% N2 (Gibco) supplement, and 5 μg/ml insulin (Sigma). For the observation of OPC differentiation, OPCs on coverslips were cultured in mOGM containing 1% FBS. After OPCs were cultured for 1 day (for proliferation assay) or 3 days (for differentiation assay) with the treatment of different concentrations of aspirin (0.1, 0.5, 1.0, 5.0, and 10 μM) and same volume of ethanol, the cells were fixed in 4% paraformaldehyde for 30 min and subjected to immunocytochemistry (see below). For Western blot, the cells at a density of 2 × 10^7^ were cultured in mOGM containing 1% FBS with 10 μM aspirin for 3 days, and then were harvested for Western blotting assay (see below).

#### RhoA activity detection

Cultured OPCs at a density of 1 × 10^7^ were maintained in mOGM containing 1% FBS, supplemented with 10 μM aspirin, 10 μM aspirin plus PD98059 [extracellular signal-related kinase (ERK) inhibitor], and the same volume of ethanol, respectively. Then, cell lysates of each group were collected and processed for Western blot as previously described (Li et al., 2013b). The activity of RhoA (Rho-GTP) was measured with the Rho Activation Assay Kit (Cytoskeleton, Inc., Denver, CO, USA), according to the protocols provided by the manufacture.

### Immunofluorescence staining

For brain tissues, the rats in different groups (*n* = 5) were sacrificed and brains were cut into 30 μm coronal floating sections through the representative corpus callosum (from the bregma 1.20 to −5.04 mm) using a Leica CM1900 cryostat (Germany). The sections were first incubated in 2 M HCl at 37°C for 30 min and neutralized with 0.1 M borate buffer (pH 8.5) for 10 min. For double immunofluorescence staining, floating sections were incubated with rat anti-BrdU (1:300, Abcam, Cambridge, UK) and mouse anti-NG2 (1:200, Millipore, Billerica, MA, USA) or rabbit anti-proteolipid protein (PLP, 1:1000, Abcam) in PBS containing 1% BSA and 0.3% Triton-X100 at 4°C overnight and then incubated with anti-rat IgG conjugated with Cy3 (1:200, Vector, Burlingame, CA, USA) or biotinylated anti-mouse/rabbit IgG (1:300, Vector) for 2 h at room temperature. After washing, the sections were reacted with Cy2-conjugated streptavidin (1:400; Jackson ImmunoResearch, West Grove, PA, USA) for 2 h at room temperature and then observed under a FV-1000 confocal microscope.

For cultured cells, the cells on the coverslips were incubated with the following primary antibodies: mouse anti-A2B5 (1:200, BD Pharmingen, Heidelberg, Germany), mouse anti-CNPase (1:500, Abcam), rabbit anti-Ki67 (1:1000, Abcam), rabbit anti-MBP (1:100, Abcam), and mouse anti-O4 (1:300, BD Pharmingen) in PBS containing 1% BSA and 0.3% Triton-X100 at 4°C overnight: after washing, the cells were incubated with anti-mouse/rabbit IgG conjugated with Cy2 or Cy3 (1:300; Vector) for 1 h at room temperature. The nuclei were stained with Hoechst 33342 (Sigma-Aldrich) for 5 min. Immunostaining signals were observed under a Leica DMIRB microscope.

### Western blot

Corpus callosum tissues and cultured cells were used for Western blotting analysis, as detailedly described by our previous study (Li et al., 2013b). Total proteins were extracted using a compartment protein extraction kit (Millipore) according to the manufacturer’s instructions. After electrophoresed on 10% SDS–polyacrylamide gels, proteins in the gels were transferred onto PVDF membranes (Millipore), which were incubated with following antibodies: anti-CNPase (1:1000, Abcam), anti-GAPDH (1:3000, Kangwei Biotechnology, Shanghai, China), anti-MBP (1:1000, Santa Cruz Biotechnology, Santa Cruz, CA, USA), and anti-PDGFαR (1:500, Abcam). After extensive washing, the membranes were incubated with horseradish peroxidase-conjugated secondary antibody (1:10000, Kangwei Biotechnology) for 1 h at room temperature and developed using an enhanced chemiluminescence western blotting detection kit (Kangwei Biotechnology). All band signals were quantified using ImageJ (NIH), and the data acquired were normalized to GAPDH expression and further normalized to the control.

### Quantification and statistical analysis

For quantitation of PLP^+^, NG2^+^, BrdU^+^/PLP^+^, and BrdU^+^/NG2^+^ cells, the immunolabeled cells in the corpus callosum were counted under the microscope at 400× magnification. The middle part of corpus callosum bordered by the bilateral cingula was chosen for counting. At least three to five independent brains were included and six coronal sections (about 300 μm apart) of each brain were used for counting or analysis. The results were expressed as the number of immunolabeled cells per square millimeter. For quantitation of cultured cells, positive cells were counted in a visual field (about 1.0 cm^2^) at 200× and the results were expressed as a percentage to the total cells indicated by Hoechst 33342 staining. At least three to five independent experiments were performed for each assay.

All data were presented as mean ± SD and analyzed using SPSS 16.0 software. Differences between groups in behavior tests and Western blot were assessed using one-way ANOVA followed by Tukey’s *post hoc* test. Differences between aspirin groups and the control *in vitro* were assessed using Student’s *t*-test. A probability of *p * < 0.05 was considered statistically significant.

## Results

### Aspirin improves animal learning and memory ability after WML

**Figure 1:**
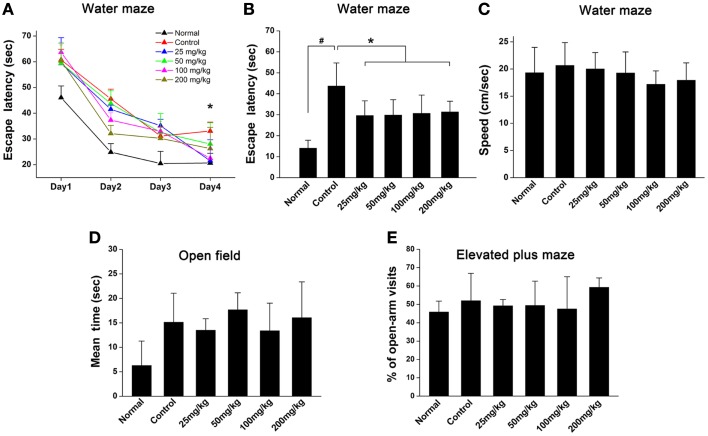
**Aspirin ameliorates cognitive impairment after WML**. **(A,B)** Morris water maze was used for the evaluation of spatial learning in rats after cerebral ischemia. **(A)** Effects of different concentrations of aspirin on escape latency (time to find the hidden platform) plotted against training day. **(B)** Escape latency recorded at fourth training day. There was no significant difference among the aspirin groups, ^#^*p * < 0.05 vs. the normal; *p  < 0.05 vs. the control. **(C)** Swimming speeds were not affected among different groups. **(D,E)** Analysis of animal anxiety-like behavior. Open field **(C)** and elevated plus maze **(D)** were used for the assessment of anxiety-like behavior.

To examine the effects of aspirin on learning and memory ability after WML in rats, Morris water maze was used to evaluate their behavior performance. Our results showed that the escape latencies in rats subjected to cerebral ischemia were significantly longer than those in normal rats (Figures 1A,B). Though rats treated with aspirin (25, 50, 100, and 200 mg/kg) were not different from those treated with ethanol controls during the 1–3 days training (Figure 1A), rats in aspirin groups took shorter time to find the platform at 4 days after training (Figure 1B). No significant difference was observed among all doses of aspirin treatment group. It was noted that there were no significant differences among rats in the various groups in swimming speed throughout four training days (Figure 1C). Additionally, it was reported that cognitive impairment was often accompanied with depression (Richard et al., 2013). To exclude possible effects of aspirin on anxiety and depression in rats, we performed open field and elevated plus maze tests. Our results showed that either the time spent in the arena center in open field (Figure 1D) or the ratio of open-arm visits in elevated plus maze test (Figure 1E), was not significantly different between the aspirin and control groups. Taken together, our behavioral results indicate that aspirin can improve learning and memory ability after WML.

### Aspirin promotes OPC proliferation and differentiation after WML

**Figure 2:**
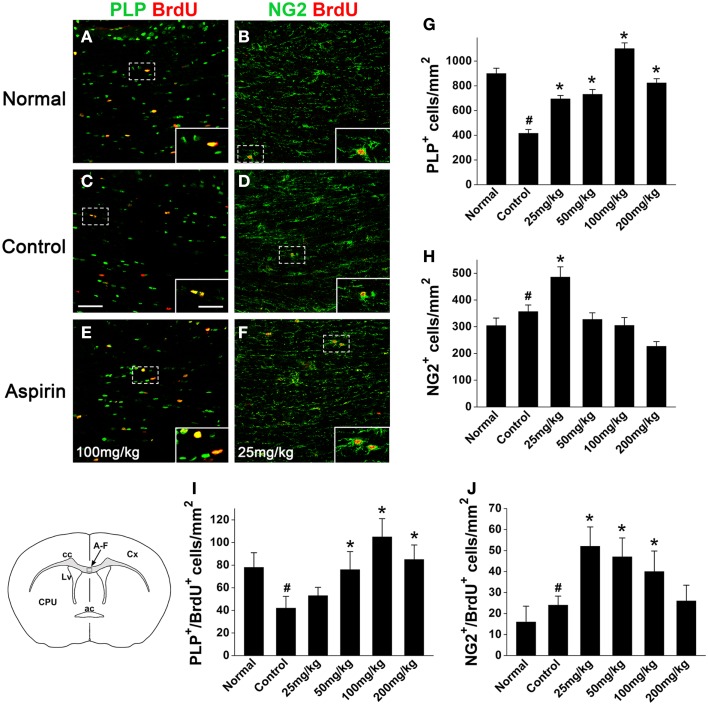
**Aspirin increases the numbers of oligodendrocytes and OPCs after WML**. **(A–F)** Representative immunostaining micrographs showing oligodendrocytes and OPCs. Coronal sections across corpus callosum (as indicated in the left lower schematic) from the normal **(A,B)**, control **(C,D)**, and aspirin-treated **(E,F)** rats were double-stained with BrdU (red) and PLP [green; **(A,C,E)**] or NG2 [green; **(B,D,F)**]. Insets show higher magnification of the cells boxed in respective panels. Scale bars: 100 μm **(A–F)**, 25 μm (insets). ac, Anterior commissure; cc, corpus callosum; Cx, cortex; CPU, caudate putamen; Lv, lateral ventricle. **(G–J)** Quantitation of the numbers of PLP^+^ **(G)**, NG2^+^ **(H)**, PLP^+^/BrdU^+^ **(I)**, and NG2^+^/BrdU^+^ **(J)** cells in each group. Note that low dose of aspirin (25 mg/kg) increased NG2-expressing cells while relatively high doses of aspirin (100–200 mg/kg) increased PLP-expressing cells, ^#^*p * < 0.05 vs. the normal; *p  < 0.05 vs. the control.

To evaluate the effects of aspirin on oligodendroglia in the WML animal brain, we examined the changes of oligodendrocyte lineage in corpus callosum at 4 weeks after cerebral ischemia. Compared with the normal rats, the number of the cells expressing PLP, a marker for mature oligodendrocyte, was significantly decreased (Figures 2A,C,G) in the control rats subjected to cerebral ischemia. However, aspirin treatment at doses of 25–200 mg/kg attenuated ischemia-induced reduction of oligodendrocytes (Figures 2C,E,G). Moreover, the number of NG2-expressing OPCs were also increased after cerebral ischemia and further elevated with treatment of 25 mg/kg aspirin whereas 50, 100, and 200 mg/kg of aspirin did not have this effect (Figures 2B,D,F,H).

**Figure 3:**
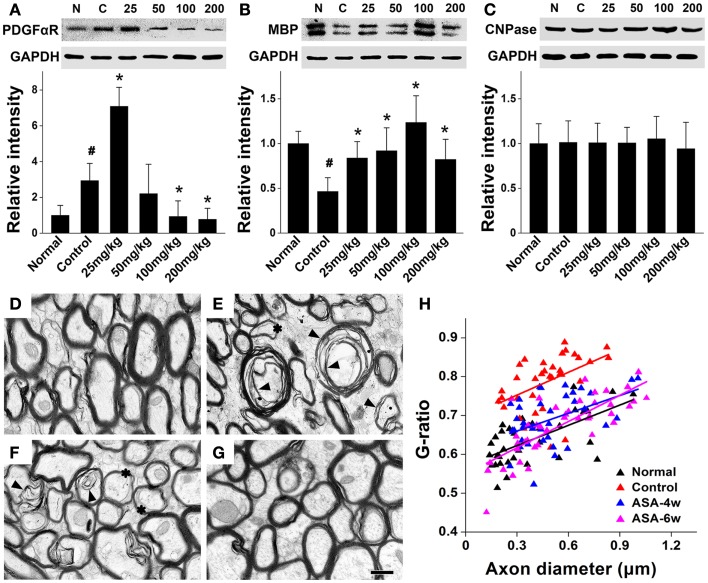
**Aspirin promotes the expression of oligodendrocyte proteins and myelin thickness after WML**. **(A–C)** Western blotting analysis of OPC protein PDGFαR **(A)**, myelin protein MBP **(B)**, and pre-oligodendrocytes protein CNPase **(C)** from corpus callosum lysates of the normal, control, and aspirin-treated rats. Note that the maximum expression levels of PDGFαR and MBP were observed in the treatment of 25 and 100 mg/kg aspirin, respectively. GAPDH was used as an internal control, ^#^*p * < 0.05 vs. the normal; *p  < 0.05 vs. the control. **(D–G)** Representative electron microscopy micrographs (40,000×) of corpus callosum from the normal **(D)**, control **(E)**, 100 mg/kg aspirin-treated group at 4 weeks **(F)**, or 6 weeks **(G)**. Arrowheads indicate demyelination and asterisks show the thinning myelin. Scale bar: 0.5 μm. **(H)** Graphical representation of the *G*-ratio of individual fibers in relation to axon diameter (presented as scatter plots) at 6 weeks. Straight lines represent the linear regression line for each dataset.

In order to investigate whether aspirin acts on the development of oligodendrocyte lineage, we examined OPC proliferation and differentiation by BrdU-pulse labeling assays. In the treatment of 100 and 25 mg/kg aspirin, the number of PLP^+^/BrdU^+^ double-stained new-differentiated oligodendrocytes and that of NG2^+^/BrdU^+^ double-labeled proliferating OPCs reached the peak (Figures 2A–F,I,J), respectively. In addition, we isolated the corpus callosum of each group and examined the protein levels of PDGFαR (a marker for OPCs), CNPase (a marker for pre-oligodendrocytes), and MBP (a marker for mature oligodendrocytes) by Western blot. Immunoblotting results showed that the expression of PDGFαR was upregulated after cerebral ischemia and further enhanced by 25 mg/kg aspirin treatment, which, however, was decreased by the treatment with 100 and 200 mg/kg aspirin (Figure 3A). By contrast, ischemia caused downregulation of MBP expression whereas 25–200 mg/kg aspirin ameliorated ischemia-induced decreased MBP expression. Noted that the MBP expression levels in 100 mg/kg aspirin-treated rats were higher than that in normal group (Figure 3B). Unexpectedly, aspirin did not affect CNPase expression (Figure 3C). Taken together, these results suggest that low dose of aspirin (25 mg/kg) may promote OPC proliferation while relatively high doses of aspirin (100 mg/kg) may enhance OPC differentiation into mature oligodendrocytes.

Furthermore, to investigate whether aspirin-induced increased mature oligodendrocytes can affect the myelin thickness after WML, we undertook an ultra-structural analysis of myelinated axons in the white matter tract of corpus callosum at 4 or 6 weeks after cerebral ischemia in rats with/without the treatment of 100 mg/kg aspirin. Compared with the normal, demyelination and reduction of myelin thickness were evident in the control rats subjected to ischemia (Figures 3D,E). Four-or six-week treatment of aspirin attenuated ischemia-induced demyelination and reduction of myelin thickness (Figures 3F,G). For quantitation, we assessed *G*-ratio, the ratio of axonal diameter to fiber diameter, which revealed an attenuated *G*-ratio in aspirin-treated rats, compared with the control (Figure 3H), suggesting a recovery in the average myelin thickness with aspirin treatment. It was noted that the effect of 6-week treatment of aspirin was seemingly better than that of 4-week treatment though with no significant difference (Figures 3F–H). Overall, our results demonstrate that depending on its dosage, aspirin may protect against ischemia-induced WML by affecting OPC fate and oligodendrocyte myelination.

### Aspirin promotes OPC proliferation and differentiation *in vitro*

**Figure 4:**
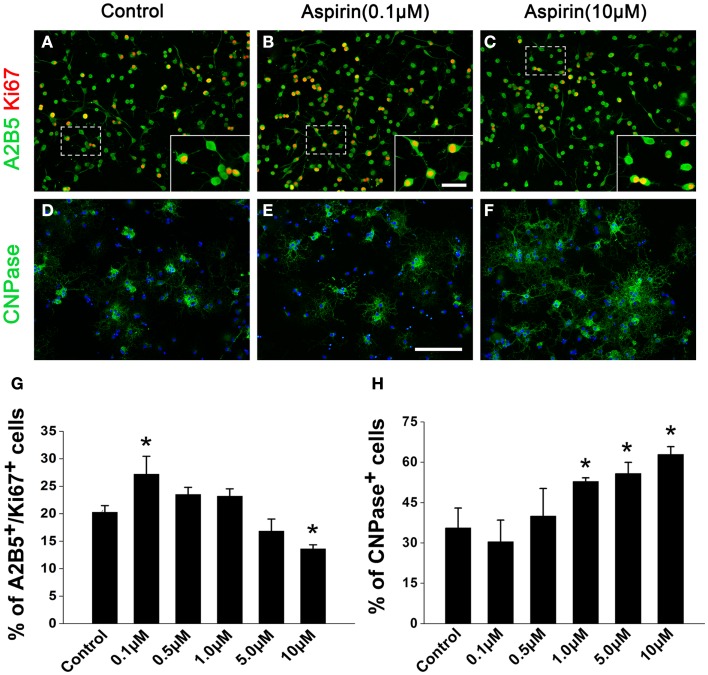
**Aspirin promotes OPC proliferation and differentiation *in vitro*. (A–F)** Representative immunostaining micrographs showing OPC differentiation. Cultured OPCs treated with ethanol (control) or different concentrations of aspirin were double-stained with Ki67 (red) and A2B5 [green; **(A–C)**] or single-stained with CNPase [green; **(D–F)**]. Nuclei were counterstained with Hoechst 33342. Insets show higher magnification of the cells boxed in respective panels. Scale bars: 50 μm **(A–F)**, 25 μm (insets). **(G,H)** Quantitation of the percentage of Ki67^+^/A2B5^+^ **(G)** and CNPase^+^ **(H)** cells to total cells in each group, *p  < 0.05 vs. the control.

In order to confirm the effects of aspirin *in vitro*, we treated cultured OPCs with different concentrations of aspirin. Double staining of A2B5 (a marker for OPCs) and Ki67 (a marker for proliferating cells) was used for the observation of OPC proliferation. Our results showed that compared with the controls, more A2B5^+^/Ki67^+^ cells were present with the treatment of 0.1 μM aspirin whereas fewer were observed with the treatment of 10 μM (Figures 4A–C,G). After OPCs were cultured in differentiation medium for 3 days, CNPase immunostaining was used for the evaluation of OPC differentiation. In the presence of 1.0–10 μM aspirin, more CNPase^+^ cells were observed than the controls while 0.1–0.5 μM did not have this effect (Figures 4D–F,H). Therefore, these *in vitro* data suggest that relatively low concentrations of aspirin could promote OPC proliferation while relatively high concentrations promote their differentiation.

### Aspirin promotes NSC differentiation into OPCs and oligodendrocytes *in vitro*

**Figure 5:**
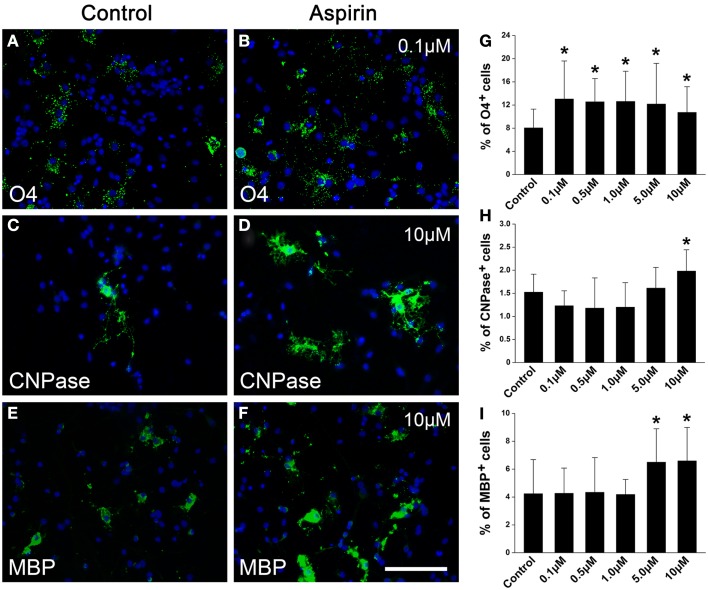
**Aspirin promotes NSC differentiation into OPCs and sequential oligodendrocytes**. **(A–F)** Representative immunostaining micrographs showing NSC differentiation. Cultured NSCs treated with ethanol (control) or different concentrations of aspirin were immunostained with O4 **(A,B)**, CNPase **(C,D)**, or MBP **(E,F)**. Nuclei were counterstained with Hoechst 33342. Scale bar: 50 μm. **(G–I)** Quantitation of the percentage of O4^+^ **(G)**, CNPase^+^ **(H)**, or MBP^+^ **(I)** cells to all cells in each group. Note that although aspirin at different concentrations increased O4-expressing cells while only relatively high concentrations of aspirin (5–10 μM) increased CNPase- or MBP-expressing cells, *p  < 0.05 vs. the control.

Since NSCs are an important endogenous source of OPCs and sequential oligodendrocytes (Gonzalez-Perez and Alvarez-Buylla, 2011), we next investigated whether aspirin could affect the differentiation of NSCs into OPCs and oligodendrocytes. NSCs cultured in a differentiation medium for 4 and 7 days were immunostained with OPC marker O4 and oligodendrocyte markers CNPase or MBP, respectively. Compared with ethanol control, aspirin with different concentrations (0.1–10 μM) increased the number of O4^+^ cells (Figures 5A,B,G). Differently, only relatively high concentrations of aspirin (5.0–10 μM) increased the number of CNPase^+^ or MBP^+^ cells, as compared to the controls (Figures 5C–F,H,I). Importantly, we found that increased number of CNPase^+^ or MBP^+^ cells companied with decreased number of GFAP^+^ cells (data not shown), indicating that the promotive effect of aspirin on NSCs differentiation into oligodendrocytes may be at the expense of astrocytes. These results imply that aspirin can promote the differentiation of NSCs into OPCs and sequential oligodendrocytes.

### Aspirin enhances ERK and inhibits RhoA activities

**Figure 6:**
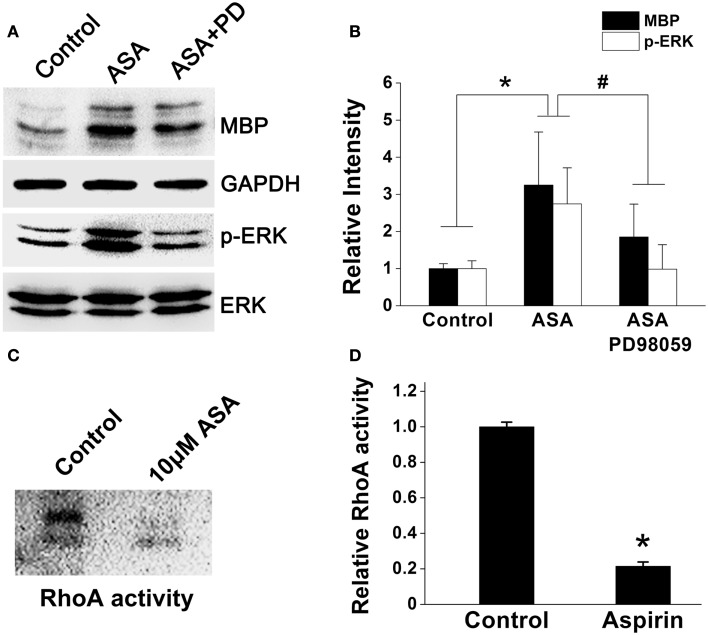
**Aspirin enhances ERK but inhibits RhoA activities**. **(A)** Western blot analysis of MBP, phospho-ERK1/2, and total-ERK1/2 protein levels from cultured OPCs treated with ethanol, 10 μM aspirin, or 10 μM aspirin plus ERK1/2 inhibitor PD98059. Note that aspirin can enhance ERK activity as revealed by phospho-ERK1/2 protein level. GAPDH was used as an internal control. **(B)** Quantitation of MBP, and phospho-ERK1/2 levels in each group, *p < 0.05 vs. the control; ^#^*p* < 0.05 vs. aspirin treatment group. **(C,D)** RhoA activity assay. Aspirin inhibited RhoA activity by approximate 80%, *p < 0.05 vs. the control.

A line of evidence has shown that p21Ras/MAPK (ERK) and Rho signaling pathways play crucial roles in oligodendroglial development (Stariha et al., 1997; Liang et al., 2004; Younes-Rapozo et al., 2009; Chew et al., 2010; Fyffe-Maricich et al., 2011; Zhao et al., 2013). Thus, to investigate the underlying mechanism(s) of aspirin, we first examined its effect on ERK1/2 pathway in OPCs cultured in a differentiation medium. Ten micromolar aspirin significantly upregulated MBP expression and enhanced ERK1/2 activity as revealed by increased phosphor-ERK expression, compared with ethanol control. However, addition of ERK inhibitor PD98059 (specific ERK kinase inhibitor; Tocris Cookson) either reversed the upregulated MBP expression and the enhanced ERK1/2 activity (Figures 6A,B). In addition, treatment of OPCs with aspirin reduced the activity of RhoA (approximate 80%), compared with the control group (Figures 6C,D). Thus, these results indicate that aspirin may promote oligodendrogenesis via enhancing ERK and inhibiting RhoA activities.

## Discussion

In the present study, we provided the first evidence that aspirin could protect against WML-induced impairment of learning and memory ability and promote OPC proliferation and differentiation. This promotive effect of aspirin may involve its ability to enhance ERK and inhibit RhoA activities.

It is known that depression is always accompanied with cognitive impairment (Richard et al., 2013). In a previous study, some NSAIDS such as aspirin or drugs like simvastatin that have an anti-inflammatory action could be useful in some depressive patients (Rahola, 2012). Furthermore, it was shown that aspirin could decrease the risk of depression in older men with high plasma homocysteine (Almeida et al., 2012). Thus, to confirm the beneficial effect of aspirin on impairment of learning and memory ability rather than depression after cerebral ischemia in the present study, we preformed open field and elevated plus maze test, and found that aspirin had no significant effects on anxiety and depression in rats.

Numerous clinical studies have shown that aspirin is widely used for stroke prevention. Depending on its dosage, aspirin has a wide spectrum of pharmacological activities and multiple sites of action. For example, its secondary preventive effects after ischemic events have been generally attributed to its antiplatelet actions through the inhibition of cyclooxygenase, which is already reached at low doses (as low as 20–40 mg/day) (Group, 1991; van Gijn, 1992). Moreover, aspirin exerts antipyretic and analgesic effects at medium doses (about 2–4 g/day) and anti-inflammatory effects at high doses (6–8 g/day) in humans (Fitzgerald, 2004). It is widely acknowledged that in humans the metabolism of many drugs may vary considerably from rodents, and therefore must be adjusted for application in rodents. Therefore, according to an equation: dose (rat)/dose (human) = BW^−0.25^ (rat)/BW^−0.25^ (human) (BW = body weight), which scales human doses to rats (Hau, 2003), we chose 25 mg/kg (relatively low dose), 100–200 mg/kg (relatively high dose) for aspirin treatment in the present study.

Application of these doses of aspirin, interestingly we found *in vivo* and *in vitro* that aspirin can promote OPC proliferation at a relatively low dose while enhance their differentiation into oligodendrocytes and myelination at a relatively high dose (Figures 2–4). We observed that low dose of aspirin can upregulate the expression of OPC markers (e.g., NG2 and PDGFαR) but meantime suppress that of mature oligodendrocyte markers (e.g., PLP and MBP). By contrast, high dose of aspirin had just the opposite effects. We proposed that more OPCs were undergoing proliferation in the treatment of low dose rather that high dose of aspirin and vice versa. Additionally, we found that aspirin seemingly had little effect on the expression of pre-mature oligodendrocytes (CNPase), and this may reflect a balance state of oligodendrocyte lineage. In addition, cell loss or dysfunction has been shown to be responsible for animal behavior deficits. It was reported that hippocampal cell loss was possibly related to memory dysfunction (Smith et al., 1991). Loss of oligodendrocytes and of myelin was involved in vascular dementia patients after hypoxic–ischemic damage (Ihara et al., 2010), whereas oligodendrocyte remyelination was strongly correlated with the recovery of cognitive dysfunction following chronic cerebral ischemia (Chida et al., 2011). In the present study, aspirin could attenuated ischemia-induced demyelination and reduction of myelin thickness (Figure 2), probably accounting for the beneficial effects of aspirin on cognitive dysfunction.

In addition to local origin, OPCs are also arise from subventricular NSCs, an important endogenous source for preserving oligodendrocytes in the white matter and for the repair of demyelinating injuries. Although under normal conditions, the production of oligodendrocytes in the SVZ is scarce (Menn et al., 2006), demyelinating lesions in the neighboring white matter can significantly increase the generation of OPCs from SVZ progenitors (Nait-Oumesmar et al., 1999; Picard-Riera et al., 2002; Menn et al., 2006), which may also account for cerebral ischemia-induced upregulated expression of OPC markers (NG2 and PDGFαR) in the present study (Figures 2 and 3). Importantly, our *in vitro* data showed that aspirin could promote the differentiation of cultured NSCs into OPCs and sequential oligodendrocytes (Figure 5), indicating a possible beneficial function of aspirin in mobilizing endogenous NSCs against WML.

A group of studies have demonstrated that the effectiveness of aspirin in stroke treatment depends on various pathways beyond its antiplatelet and anti-inflammatory functions. Low dose aspirin was reported to inhibit the activity of RhoA (Li et al., 2013a), one of well-known members of the Rho GTPase family that regulates a wide range of fundamental cell functions such as proliferation and apoptosis. RhoA inactivation was found to enhance the proliferation of hematopoietic stem and progenitor cells (Ghiaur et al., 2006) and the differentiation and myelination in oligodendrocytes (Zhao et al., 2013). In addition, ERK pathways have particularly well-documented roles in proliferation and differentiation for various stem and precursor cells (Burdon et al., 2002). Constitutive activation of Erk1/2 signaling in oligodendrocytes not only caused myelination to occur earlier but also significantly induced more oligodendrocytes to myelinated *in vitro* (Xiao et al., 2012). Consistently, our *in vitro* results showed that aspirin could enhance ERK but inhibit RhoA activities (Figure 6), indicating aspirin-mediated ERK activation and RhoA inhibition may be involved in OPC proliferation and differentiation. Intriguingly, a group of studies had shown that aspirin exerted an inhibitory effect on ERK activity in neuronal protection or apoptosis induction of carcinoma cells (Tegeder et al., 2001; Vartiainen et al., 2003; Park et al., 2010; Im and Jang, 2012). It is likely that this discrepancy of aspirin effectiveness may depend on different cell types and injury models used since these studies mainly focused on the effects of ERK on apoptosis during inflammation and tumorigenesis. By contrast, our study engaged in the effects of ERK on proliferation and differentiation during oligodendrocyte development.

In summary, we provide the first evidence that aspirin can promote OPC proliferation and differentiation and oligodendrocyte myelination after chronic cerebral ischemia by at least activating ERK and inhibiting RhoA activities. This promotive effect of aspirin on oligodendrocyte lineage may account for the recovery of ischemia-induced cognition impairment, suggesting a promising role of aspirin in the treatment of WML.

## Conflict of Interest Statement

The authors declare that the research was conducted in the absence of any commercial or financial relationships that could be construed as a potential conflict of interest.
